# Quantification of fluorophore distribution and therapeutic response in matched *in vivo* and *ex vivo* pancreatic cancer model systems

**DOI:** 10.1371/journal.pone.0229407

**Published:** 2020-02-25

**Authors:** Allison Solanki, Diana King, Guillaume Thibault, Lei Wang, Summer L. Gibbs

**Affiliations:** 1 Department of Biomedical Engineering, Oregon Health & Science University, Portland, Oregon, United States of America; 2 Knight Cancer Institute, Oregon Health & Science University, Portland, Oregon, United States of America; 3 Center for Spatial Systems Biomedicine, Oregon Health & Science University, Portland, Oregon, United States of America; Columbia University, UNITED STATES

## Abstract

Therapeutic resistance plagues cancer outcomes, challenging treatment particularly in aggressive disease. A unique method to decipher drug interactions with their targets and inform therapy is to employ fluorescence-based screening tools; however, to implement productive screening assays, adequate model systems must be developed. Patient-derived pancreatic cancer models (e.g., cell culture, patient-derived xenograft mouse models, and organoids) have been traditionally utilized to predict personalized therapeutic response. However, cost, long read out times and the inability to fully recapitulate the tumor microenvironment have rendered most models incompatible with clinical decision making for pancreatic ductal adenocarcinoma (PDAC) patients. Tumor explant cultures, where patient tissue can be kept viable for up to weeks, have garnered interest as a platform for delivering personalized therapeutic prediction on a clinically relevant timeline. To fully explore this *ex vivo* platform, a series of studies were completed to quantitatively compare *in vivo* models with tumor explants, examining gemcitabine therapeutic efficacy, small molecule uptake and drug-target engagement using a novel fluorescently-labeled gemcitabine conjugate. This initial work shows promise for patient-specific therapeutic selection, where tumor explant drug distribution and response recapitulated the *in vivo* behavior and could provide a valuable platform for understanding mechanisms of therapeutic response and resistance.

## Introduction

Therapeutic resistance is a common phenomenon plaguing both initial cancer therapy as well as overall treatment response due to acquired resistance to the selected treatment regimens. Additionally, some cancers remain occult until late stages, such as pancreatic ductal adenocarcinoma (PDAC), the most common of all pancreatic cancers, where the approved treatment options present little efficacy against these complex tumors. Due to this challenge, five-year survival rates for PDAC still average in the single digits [1, 2]. PDAC hallmarks, including heterogeneous genetic abnormalities, suppressed immune response, metabolic resistance, and the acquired morphology of the diseased tissue (e.g., dense desmoplasia), have all been blamed for innate and induced therapy resistance [3, 4]. Efforts to combat these treatment obstacles, including unveiling genetic vulnerabilities in the disease (e.g., *KRAS*, *TP53*, and *SMAD4*) and use of the only approved targeted therapy, erlotinib, have yielded only marginal success [5–7]. Thus, new methods to elucidate the mechanisms behind the failure of promising treatment regimens in patient specific model systems is imperative.

A remaining concern is whether the applied therapeutic reaches its intended tumor target, a repercussion of a robust self-defense mechanism that tumor cells and the surrounding microenvironment put forth [8]. Uncoupling these complex tissue-drug interactions have been analyzed in the context of overall drug distribution using radiolabeled therapeutics [9, 10], mass spectroscopy tissue analysis [11, 12] and computational modeling [13, 14], however these tools have limited utility for extensive, patient-specific screening assays to inform personalized therapy. Although fluorescent-based assays have been gaining traction [15], a patient-specific screening assay has yet to be developed. In this work, we sought out patient-specific model systems that would provide both a rapid read out to guide therapy and would recapitulate drug distribution behavior *in vivo*. Patient-derived pancreatic cancer models (e.g., cell culture, patient-derived xenograft [PDX], genetically engineered mouse models [GEMM], and organoids) have been utilized extensively to study PDAC and predict therapeutic viability [16, 17]. However, cost, long read out times and the inability to fully recapitulate the tumor microenvironment have rendered most models incompatible with clinical decision making for PDAC patients. Tumor explant cultures, in which patient tissue is kept viable in optimized culture conditions, have garnered interest as a platform for delivering personalized therapeutic prediction on a clinically relevant timeline. Explant samples are routinely reported to be viable for up to a week in culture using standard laboratory culture settings. Numerous studies have detailed maintained tissues viability for sufficient periods to extract crucial therapeutic response information for various cancers [11, 18–21], including PDAC [22–26]. However, to date no study has directly examined drug-target interaction in tumor explants. Herein, we assessed the utility of the explant culture system to quantify drug-tumor target interactions with the potential to enhance our understanding of therapeutic response and resistance mechanisms.

As a proof-of-concept, we hypothesized that far-red, cell membrane permeable fluorophores with low tissue accumulation would be ideal candidates for synthesis of labeled drug conjugates, allowing for visualization in heterogeneous patient-derived explant tissues. Furthermore, we predicted that tumor explants would be a suitable system for studying the spatial localization of fluorescently labeled drug compounds, thus providing an exciting new pathway for understanding limitations in current treatment options for PDAC and other cancers. To fully explore whether treated *ex vivo* tumor explant cultures accurately reflect the drug-tissue interactions of *in vivo* administration, we quantitatively compared fluorescent drug accumulation with matched tumor explant samples. Both heterotopic cell-derived xenograft (CDX) and PDX mouse models were used for these initial experiments, as matched *in vivo* specimens were readily attainable. Although similar tests have been conducted previously in other cancer systems [27], none, to our knowledge, have compared different PDAC model systems in the context of a fluorescently labeled drug platform. We focused our work on the chemotherapeutic gemcitabine, but envision expansion of our fluorescently-conjugated drug toolbox to other cancer therapeutics. Gemcitabine is a nucleoside analog prodrug that halts DNA synthesis and has been administered as a single or combination therapy to PDAC patients for over 20 years with varying success, and remains the first-line of treatment for many patients [28]. In the presented study, the parent drug was administered in both *in vivo* and *ex vivo* environments, verifying that tissue response was consistent in both systems via quantified immunofluorescence. *Ex vivo* small molecule uptake studies were conducted, confirming an appropriate fluorophore candidate for drug labeling, where low tissue accumulation was considered the metric for success. Titration experiments were completed in matched tumor explant and *in vivo* tumor tissues to confirm minimal non-specific uptake of the parent fluorophore of choice, Atto680, a zwitterionic oxazine-based fluorophore. These studies informed a culminating study investigating the uptake of gemcitabine that was labeled with Atto680, where distinctive, matched tissue uptake patterns were seen *in vivo* and *ex vivo* with the greatest accumulation coincident with necrotic tissues. This exciting finding could be readily extended to other drugs and cancer types to examine drug-tissue uptake and accumulation patterns in the context of therapeutic response and resistance.

## Materials and methods

### Synthesis of fluorescently labeled gemcitabine

**Fig 1:**
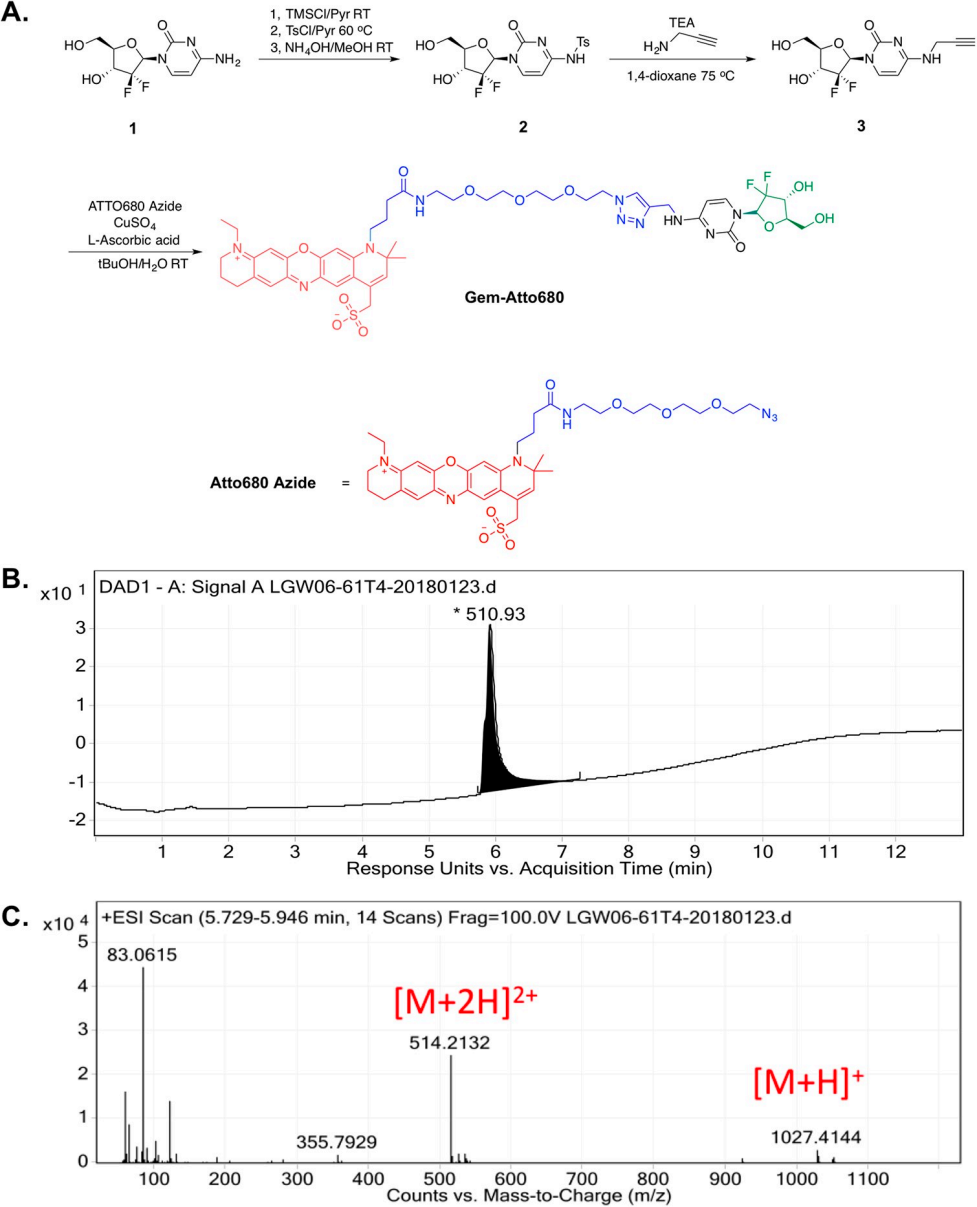
Synthesis of fluorescently labeled gemcitabine (Gem). **A**. Gemcitabine (Compound **1**) was covalently labeled at the 4-amino group using the Atto680 fluorophore (red) through an inert linker (blue). The portion of the parent gemcitabine molecule most important for biological activity is labeled in green. TMSCl = Trimethylsilyl chloride; Pyr = pyridine; RT = room temperature; TsCL = *p*-Toluenesulfonyle chloride; MeOH = methanol; TEA = triethylamine; *t*BuOH = *tert*-butanol. LC-MS was used to verify the purity of fluorescently labeled Gem-Atto via **B**. area under the curve analysis of the absorbance at 254 nm and **C**. mass to charge (m/z) ratio in positive ion mode. Reaction yields were as follows: Compound **2** (83%); Compound **3** (48%); Gem-Atto (78%).

All reagents were purchased from Sigma-Aldrich (St. Louis, MO), Thermo Fisher Scientific (Waltham, MA), TCI America (Portland, OR) or Combi-Blocks Inc. (San Diego, CA). Atto680 azide was purchased from ATTO-TEC (Siegen, Germany). Unless otherwise indicated, all commercially available starting materials were used directly without further purification. Analytical thin layer chromatography (TLC) was performed on Millipore ready-to-use plates with silica gel 60 (F254, 32–63 μm, EMD Millipore, Burlington, MA). Flash chromatography was performed on Sorbent Technologies silica gel (Sorbent Technologies Inc., Norcross, GA) for column chromatography. High-resolution mass spectra (HRMS) were measured on an Agilent 6244 time-of-flight tandem liquid chromatography mass spectroscopy (LC-MS) instrument with a diode array detector VL+ (Agilent Technologies, Santa Clara, CA). The synthesis of gemcitabine labeled with Atto680 is detailed below (Fig 1A).

### *N*-(1-((2*R*,4*R*,5*R*)-3,3-difluoro-4-hydroxy-5-(hydroxymethyl)tetrahydrofuran-2-yl)-2-oxo-1,2-dihydropyrimidin-4-yl)-4-methylbenzenesulfonamide (2)

Compound **2** was synthesized using a slight modification of the previously published protocol [29]. Compound **1** (gemcitabine, 300 mg, 1 mmol, Acros Organics, product number 461060050, lot number A0375170) was suspended in 5 ml anhydrous pyridine under nitrogen (N_2_), to which trimethylsilyl chloride (1.27 ml, 10 mmol, Sigma-Aldrich, product number 386529-100ML, Lot number SHBH8549) was added. The reaction mixture was stirred at room temperature for 2 hours, followed by addition of *p*-toluenesulfonyl chloride (1.91 g, 10 mmol, Acros Organics, product number 139021000, lot number A0334970). The resulting reaction mixture was stirred for an additional 24 hours at 60 °C. After cooling to room temperature, the solvent was removed under reduced pressure. The resulting residue was resuspended in 10 ml ammonium hydroxide (NH_4_OH)/Methanol (MeOH) (1/9, v/v) solvent mixture and stirred at room temperature for 24 hours. The reaction mixture was concentrated *in vacuo*, and purified by flash column chromatography with silica gel (25 g), using ethyl acetate (EtOAc)/hexanes as eluent to give compound **2** (0.348 g, 83%) as a white solid.

### 1-((2*R*,4*R*,5*R*)-3,3-difluoro-4-hydroxy-5-(hydroxymethyl)tetrahydrofuran-2-yl)-4-(prop-2-yn-1-ylamino)pyrimidin-2(1*H*)-one (3)

To a microwave vial, compound **2** (0.21 g, 0.503 mmol), propargylamine (0.658 ml, 10.06 mmol, Combi-Blocks, product number OS-7456, lot number B27341), and triethylamine (2.10 ml, 15.1 mmol) were dissolved in 5 ml 1,4-dioxane. The tube was sealed with a Teflon cap, and the reaction mixture was heated to 75 °C and stirred for 3 days. After cooling to room temperature, the volatiles were removed *in vacuo*, and the resulting residue was purified by flash column chromatography with silica gel (25 g), using MeOH/EtOAc as eluent to give compound **3** (72 mg, 48%).

### (11-(1-(4-(((1-((2*S*,4*R*,5*R*)-3,3-difluoro-4-hydroxy-5-(hydroxymethyl)tetrahydrofuran-2-yl)-2-oxo-1,2-dihydropyrimidin-4-yl)amino)methyl)-1*H*-1,2,3-triazol-1-yl)-13-oxo-3,6,9-trioxa-12-azahexadecan-16-yl)-1-ethyl-10,10-dimethyl-3,4,10,11-tetrahydro-2*H*-dipyrido[3,2-*b*:2',3'-*i*]phenoxazin-1-ium-8-yl)methanesulfonate (Gem-Atto)

Under N_2_, compound **3** (1 mg, 3.33 μmol) and Atto680 azide (2 mg, 2.38 μmol, ATTO-TEC, product number AD 680–101, lot number SA10W25F6) were suspended in 2 ml degassed *tert*-butanol/H_2_O (1/1, v/v). Freshly prepared 100 mM L-ascorbic acid solution (35 μl, Sigma-Aldrich, product number A4034-100G, lot number SLBR2743V) and copper sulfate (CuSO_4_) solution (15 μl, Acros Organics, product number 197722500, lot number A0345629) were added to the solution. The resulting reaction mixture was stirred at room temperature overnight and purified using preparative high-performance liquid chromatography (HPLC, Agilent 1250 Infinity HPLC) with a C18 column (150 × 21.2 mm). The sample was eluted using solvents A: water-formic acid (99.9:0.1, v/v) and B: acetonitrile-formic acid (99.9:0.1, v/v), with the gradient increased from 10% B to 50% B over 30 min, and from 50% B to 90% B over 5 min at a flowrate of 10 ml/min. The fractions containing product were frozen and lyophilized to afford Gem-Atto (2 mg, 78%) as a blue solid. HRMS (ESI-TOF) m/z [M+H]^+^ calculated for C_47_H_60_F_2_N_10_O_12_S: 1027.4154, found: 1027.4144; m/z [M+2H]^2+^ calculated for C_47_H_60_F_2_N_10_O_12_S: 514.2113, found: 514.2132 (Fig 1B).

### Cell lines

The human pancreatic cancer cell lines used in this study, PANC-1, AsPC-1 and Capan-1, were generously provided by Dr. Rosalie Sears’ laboratory at Oregon Health and Science University (OHSU). The cells were originally purchased from ATCC (Manassas, VA) and maintained at passage numbers <25 throughout experimentation. The cell lines were expanded in their respective growth media (PANC-1, AsPC-1: DMEM [Thermo Fisher Scientific] + 10% fetal bovine serum [FBS, VWR Scientific, Radnor, PA] + 1% penicillin/streptomycin/glutamine [Thermo Fisher Scientific]; Capan-1: RPMI 1640 [Thermo Fisher Scientific] + 10% FBS + 1% penicillin/streptomycin/glutamine) and stored in a 37 °C, 5% CO_2_ incubator.

### Functional assessment of Gem-Atto

Changes to drug target binding were quantified using a competitive binding assay between Gem-Atto and the parent drug, gemcitabine, on the established human pancreatic cancer cell lines: PANC-1, AsPC-1 and Capan-1. The cells were washed, trypsinized and plated in a 96-well glass bottom plate (Cellvis, Mountain View, CA) at a concentration of 0.1x10^6^ cells/ml. The cells equilibrated for 24 hours after which, gemcitabine-HCl (Sigma-Aldrich) was added to all cell lines at 0, 0.005, 0.05, 0.5, 5, 50 μM in triplicate wells. Every well also contained Gem-Atto at a single concentration (500 nM). Following a 24-hour incubation, the cells were gently washed 3 x 5 min with phosphate buffered saline (PBS), fixed with 4% paraformaldehyde (PFA) for 15 min, washed again with PBS for 5 min and imaged in fresh PBS on a Zeiss AxioObserver inverted fluorescence microscope (Carl Zeiss, Oberkochen, Germany). For fluorescence excitation, a PhotoFluor II broad band light source (89 North, Burlington, VT) was filtered using a 650 ± 22.5 nm bandpass excitation filter and a 720 ± 30 nm bandpass emission filter to acquire images for Atto680. Individual cellular fluorescence was quantified using FIJI and analyzed with Prism (GraphPad, San Diego, CA).

### Animal care

All animal experiments were approved by the OHSU Institutional Animal Care and Use Committee (IACUC). All mice were housed in the OHSU vivarium, an IACUC and AAALAC approved facility, and supplied with standard food, water and daily inspection to ensure that they were not under pain or distress throughout the duration of the experiments. Mice were placed on a chlorophyll-free diet one week prior to tumor resection (Animal Specialties, Inc, Hubbard, OR). All rodent surgical procedures, detail below, were carried out under full anesthesia utilizing a 90/10 mixture of ketamine/xylazine. Ketamine was administered at a dose of 100 mg/kg (Hospira Inc., Lake Forest, IL) and xylazine was administered at a dose of 10 mg/kg (AnaSed, Shenandoah, IA) by intraperitoneal (IP) injection. Depth of anesthesia was verified using the toe pinch method to ensure lack of reactivity and that rodents were fully anesthetized prior to commencement of any surgical procedures. The routine method of euthanasia for mice was inhalation of carbon dioxide under full anesthesia at the end of each experiment. Euthanasia of all mice was confirmed by physical examination to ensure heartbeat and respiration had ceased. This method of euthanasia is humane and rapid and is consistent with the recommendations of the Panel on Euthanasia of the American Veterinary Medical Association.

### Mouse PDAC xenograft models

Athymic nude mice (Homozygous 490, Charles River Laboratories, Wilmington, MA) were implanted with PANC-1, AsPC-1 or Capan-1 cells, which were expanded *in vitro* using their respective growth media. The cultured cells were trypsinized, counted and resuspended in growth media to a concentration of 2 x 10^7^ cells/ml. Anesthetized mice were implanted with cells into each rear flank at a final concentration of 1 x 10^6^ cells/flank in 50% v/v Matrigel (Corning Inc., Corning, NY), resulting in two tumors/mouse. For implantation, the syringe was inserted and held in place for 10 sec following completion of the injection to prevent any cell suspension leakage. Mice were monitored daily after implantation for tumor growth. The tumors were allowed to grow until they reached the appropriate size for each respective experiment (100–1000 mm^3^, see below). A total of n = 68 athymic nude mice were implanted for CDX model generation and utilized for *in vivo* experiments as well as generation of tumor explant tissues. This total mouse number includes five additional mice to account for an 80% PANC-1 tumor implantation take-rate.

Immunocompromised mice NSG(NOD, Cg-Prkdc^scid^ Il2rg^tm1Wjl^/SzJ) mice (Jackson Laboratory, Bar Harbor, ME), housed in a modified barrier facility, were implanted with patient biopsy tumor pieces (passage 3, gift from the laboratory of Dr. Rosalie Sears, OHSU) from a female patient, age 52, presenting with metastatic PDAC that was identified as initially responsive to gemcitabine treatment. A total of n = 15 NSG mice were utilized for tissue expansion, and an additional n = 12 mice were used for *in vivo* experiments, including control and tumor explant tissue generation. Briefly, frozen tissue was thawed in freezing media (90% FBS + 10% dimethyl sulfoxide [DMSO]) rapidly just prior to implantation. Under sterile conditions, a small incision was made on the shoulder flank of each anesthetized mouse. After tenting the skin, a single tumor sample (~5x5 mm^2^) was dipped in Matrigel and then inserted beneath the skin at the incision site. The incision was closed with suture and the mouse was allowed to recover under observation.

### *In vivo* assessment of fluorophore biodistribution and treatment response

When tumor size was between 100–200 mm^3^, a subset of tumor bearing mice were selected to assess response to gemcitabine. Tumor size was measured and recorded prior to each gemcitabine administration using digital calipers. Following tumor size measurements, 100 mg/kg gemcitabine-HCl was administered IP on days 0, 3, 6, and 9 [30, 31]. The mice were euthanized 2 hours after the final gemcitabine dose on day 9 of the treatment study. The tumor tissue was harvested and immediately placed into optimal cutting temperature (OCT, Fisher Scientific, Hampton, NH) compound and frozen for further analysis.

The mice with tumors measuring approximately 1000 mm^3^ were administered 1.25 or 2.50 mg of Atto 680-carboxylic acid (Atto680), gemcitabine-Atto680 (Gem-Atto), or vehicle IP. Atto680 and Gem-Atto were reconstituted at 10 mM in DMSO and diluted to a final concentration of 10 mg/ml in saline for IP injection. The mice were euthanized 4 hours after IP administration. The tumor tissue was harvested and snap frozen in OCT for further analysis. The frozen blocks were cryosectioned at 10 μm thickness and stored at -80 °C until they were mounted with Fluoromount-G (Southern BioTech, Sigma-Aldrich) and coverslipped. Full tissue fluorescence images were acquired on a Zeiss AxioScan.Z1 slide scanner with a 10x 0.45 PlanApo objective (Zeiss) using an exposure time of 2500 ms with excitation and emission filters at 640/30x and 690/50m, respectively, for image collection. The acquired 10x images were tiled into a full tissue fluorescence image using Zen software (Zeiss).

### *Ex vivo* assessment of fluorophore biodistribution and treatment response

Xenografts from patient-derived tissues and cell lines were grown to a maximum size of 1000 mm^3^ prior to harvest for *ex vivo* tumor explant culture. Upon resection, tumor tissue was placed in explant media (RPMI 1640 supplemented with 10% FBS, 1 mg/100 ml hydrocortisone [Sigma-Aldrich], and 1x antimycotic/antibiotic [Sigma-Aldrich]) and transported on ice for explant preparation. Under sterile conditions, tumor tissue was subdivided into small fragments (~1x2x2 mm) using surgical scalpels within one hour of tumor resection. Individual tissue slices were plated onto trans-well inserts (EMD Millipore, Burlington, MA) equilibrated in pre-warmed tumor explant media in 6-well plates. Explants were coated with one drop of optimized explant media to ensure tissue hydration. Plates were then placed in a 37 °C, CO_2_ incubator and allowed to equilibrate for 24 hours prior to incubation with any reagent containing media.

For treatment response studies, explanted tissues were incubated with 0, 0.5, 1, 5, or 5 μM of gemcitabine, reconstituted in optimized explant media, for 48 hours. All treated explants were frozen in OCT, cryosectioned at 10 μm thickness and stored at -80 °C until further analysis.

For fluorophore biodistribution studies, explanted tissues were incubated with different carboxylic acid terminated fluorophores including Alexa Fluor 488 (AF488), Alexa Fluor 546 (AF546), Alexa Fluor 647 (AF647, Thermo Fisher Scientific, Waltham, MA) and Atto680 (ATTO-TEC). The fluorophores were reconstituted at 10 mM in DMSO, then further diluted to 500 nM in 1x PBS, pH 7.4 for working experimental stocks. The fluorophores were incubated with the tissue explants at 500 nM and tissue samples were collected after 72 hours of total incubation time. All explanted tissue samples were frozen in OCT and cryosectioned at 10 μm thickness. Full tissue fluorescence images were acquired on a Zeiss AxioScan.Z1 slide scanner with a 10x0.45 PlanApo objective using the following exposure times as well as excitation (x) and emission (m) filter combinations for image collection: AF488—2500ms, 470/40x, 525/50m; AF546—500ms, 550/25x, 605/70m; AF647/Atto680—2500ms, 640/30x, 690/50m.

### Immunofluorescence (IF) assays

N-Hydroxysuccinimide (NHS) ester functionalized fluorophores including AF488, AF647, and Cy3B (GE Healthcare, Marlborough, MA) were purchased for antibody labeling using standard NHS ester chemistry. All fluorophore conjugated antibodies were purified via size exclusion chromatography on a Bio-Scale Mini Bio-Gel P-6 Cartridge (NGC Quest Plus 10, Bio-Rad, Hercules, CA) into 1x PBS, pH 7.4. The antibody-fluorophore conjugates were concentrated with a 0.5 ml Amicon centrifugal filter unit (MWCO = 10 kDa, EMD Millipore). The final antibody concentration and conjugation ratios (fluorophore:antibody) were calculated using spectrophotometry (NanoDrop, Thermo Fisher Scientific). Conjugation ratios were kept within a range of 1.1–3.3 for all antibody conjugates. Ki67 (polyclonal, Abcam, Cambridge, MA) was directly conjugated to AF647 (conjugation ratio = 1.1) and stained at final concentration of 20 μg/ml. Beclin 1 (G-11, monoclonal, Santa Cruz Biotechnology, Dallas, TX) was directly conjugated to Cy3B (conjugation ratio = 2.9) and stained at a final concentration of 10 μg/ml. Cleaved Caspase-3 (CC-3, Asp175, 5A1E, monoclonal, Cell Signaling Technology, Danvers, MA) was indirectly labeled using donkey anti-rabbit secondary antibody (Jackson ImmunoResearch, West Grove PA) conjugated to AF488 (conjugation ratio = 3.3). CC-3 was stained at a dilution of 1:100 from the stock antibody solution. Nuclei were stained using DAPI (300 nM final concentration, Sigma-Aldrich).

hENT1 quantification of adherent, fixed PANC-1, AsPC-1 and Capan-1 cells was performed using the ENT1 (F12, monoclonal, Santa Cruz Biotechnology) primary antibody and visualized with a donkey anti-mouse secondary antibody (Jackson ImmunoResearch) conjugated to Cy3B (conjugation ratio = 2.8).

For IF assessment of tissue viability, frozen tissue sections were fixed briefly in 4% PFA prior to the staining. After antibody staining, tissue sections were fixed again in 4% PFA prior to Fluoromount-G application and coverslipping. Four tissue sections were quantified for each experimental condition to assess intratumoral tissue heterogeneity. Antibody stained tissues were imaged at 20x magnification on a Zeiss AxioScan.Z1 slide scanner. All cohorts were imaged sequentially with a secondary only control slide, which was subsequently used to normalize all stained images for quantification purposes and correct for autofluorescence or other artifacts.

IF viability staining was quantified using custom written software to tabulate total cell count based on the DAPI staining. Antibody staining was next segmented on a cell-by-cell basis and positive nuclear overlap was counted in order to determine the total number of individual positive staining events. The image segmentation pipeline was primarily composed of mathematical morphology operations [32]. The image contrast was first normalized using contrast limited adaptive histogram equalization (CLAHE), then background was subtracted with a white top-hat function, where the structuring element radius was defined by the theoretical nuclei radii. Next, an ultimate opening found each nucleus center, including the touching nuclei. The nuclei centers were then used in a seeded watershed to segment cells. Any defined artifacts and nuclear clusters were removed from the final analysis. From the nuclei segmentation, a Voronoi diagram was computed to allow for distance map computation.

### Fluorescence intensity image quantification

Tissues from mice administered fluorophore *in vivo* and explants incubated with fluorophores were analyzed for overall fluorescence intensity using custom written MatLab code (MathWorks, Natick, MA). One additional serial section was reserved for morphological analysis using standard hematoxylin and eosin (H&E) staining to differentiate between viable tumor and necrotic tissue regions. Delineated regions of viable and necrotic tissue were quantified independently for fluorescence intensity to determine whether fluorophore uptake was correlated to tissue viability. These regions of interest (ROIs) were selected to encapsulate the entire tissue except for an ~10 μm border around the edge of the tissue, as well as any apparent folds or cutting artifacts. An average fluorescence signal was calculated by normalizing the overall fluorescence intensity by the total number of pixels contained within the ROI. Control tissue (uninjected or unstained) was analyzed separately and fluorescence intensity computed for parallel analysis with all fluorophore treated samples.

### Statistical analysis

All data presented herein is plotted as the mean with the standard error of the mean (SEM) and statistical significance was analyzed using one-way ANOVA tests, unless otherwise noted. An average of 10 ROIs per imaging dataset containing a minimum of 5000 total nuclei were analyzed for every experimental cohort.

## Results and discussion

### Tumor explant viability vs. *in vivo* tumor growth

Tumor explant culture provides a methodology to screen potential cancer therapeutics in a clinically relevant time frame using intact patient tissues, offering the potential for personalized medicine through quantification of tissue-drug interactions. Herein, two PDAC model systems were utilized to enumerate the relationship between *in vivo* and *ex vivo* administration of small molecule therapeutics. Initial studies were completed using human pancreatic cancer CDX models with varying levels of gemcitabine resistance (AsPC-1, PANC-1, and Capan-1) and then translated to a gemcitabine sensitive PDX to model the potential for clinical utility of the explant culture system. Comparison across both xenograft model systems provided a unique opportunity to study small molecule uptake in heterogeneous patient-derived tumors versus homogenous tumor populations derived from established cancer cell lines. Although xenografts from established cancer cell lines lack the extensive stromal tissue known to be a hallmark of human PDAC, these models were used as a simplified system to establish the relationship between small molecule distribution differences *in vivo* vs. *ex vivo* before moving to the more complex patient-derived tissue systems.

**Fig 2:**
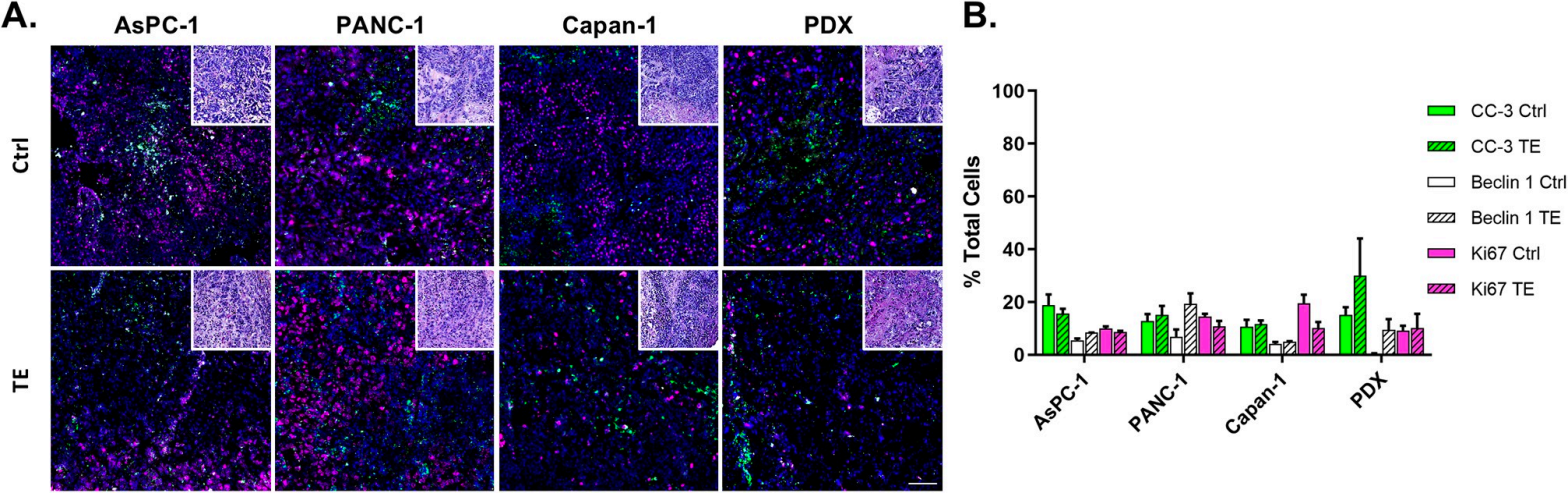
Tumor explant (TE) viability. The IF viability panel (Ki67, CC-3 and Beclin 1) was used to stain four 10 μm tissue sections per time point following tumor resection. **A**. Representative IF images of tumor tissue sections immediately after resection (Ctrl, top row) or following 72 hours in explant culture (TE, bottom row) are shown false colored as follows: DAPI (nuclear marker, blue), Cleaved Caspase-3 (CC-3, cytosolic staining marking apoptosis, green), Beclin 1 (cytosolic staining marking autophagy, white), Ki67 (nuclear stain marking proliferation, pink). Corresponding H&E images of the preserved tissue morphology are also shown (inset, upper right). Scale bar = 100μm. **B**. Tissue viability was quantified by assessing positive antibody staining and used to calculate the percentage of stained cells at each time point per condition. N = 3 athymic nude mice (two tumors/mouse) were used per CDX model to provide sufficient starting material for control and treated TE tissue samples.

Initial studies were completed to confirm tumor explant viability in culture, where viability for up to five days was confirmed by IF. From these studies, the 72-hour time point was selected for further study as it facilitated explant preparation, culture, treatment and analysis within one week of tissue resection for future clinical translation. Notably, quantified explanted tissue viability mirrored results from *in vivo* samples. AsPC-1, PANC-1, Capan-1 and PDX tumor explants maintained their morphology and displayed similar proliferation and cellular death profiles as their *in vivo* control counterparts (Fig 2A). Analysis of a minimum of 5000 nuclei per experimental condition showed that CC-3, Beclin 1 and Ki67 staining occurred in ~10–20% of the total cell populations in all model systems for each antibody (Fig 2B). Notably, cellular proliferation was well dispersed for both *in vivo* and *ex vivo* samples, demonstrating viability of the tumor tissue when grown in explant culture.

### Gemcitabine sensitivity of CDX & PDX PDAC models

**Fig 3:**
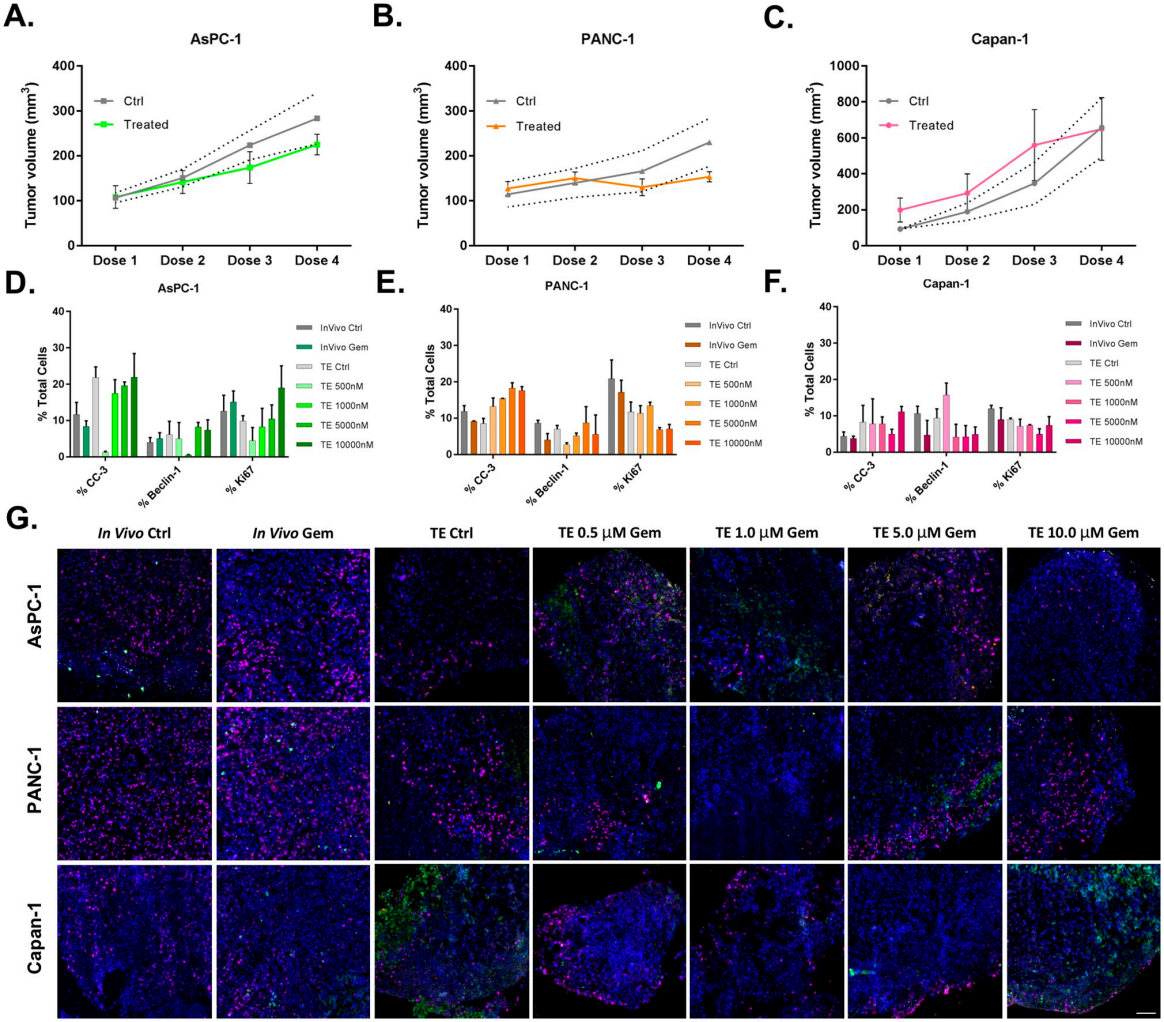
Gemcitabine (Gem) treatment equivalence in PDAC xenograft cell line models *in vivo* vs. *ex vivo*. Mice bearing xenografts established from three PDAC cells lines were treated with 100 mg/kg gemcitabine IP on days 0, 3, 6 and 9 to model the clinical dose and administration schedule. Tumor volumes were quantified before and during treatment for both vehicle (Ctrl) and gemcitabine treated **A**. AsPC-1, **B**. PANC-1 and **C**. Capan-1 tumors as a measure of treatment response. The standard deviation in the tumor volume of untreated control samples is plotted as dashed gray lines. *Ex vivo* explants (TE) grown from untreated xenograft tumors were treated with gemcitabine (0.5–10 μM) in culture for 48 hours. All resected tumor tissue (*in vivo* or *ex vivo*) was fresh frozen following the final dose of gemcitabine. Serially sectioned tissues were stained with the IF viability panel to assess biological response, where at least two 10 μm sections per condition were quantified for the percentage of positive cells for **D**. AsPC-1, **E**. PANC-1 and **F**. Capan-1 CDX tumors treated *in vivo* or *ex vivo* in explant culture. **G**. Representative IF viability panel images from untreated and gemcitabine treated tissues are shown with staining false colored as DAPI (all nuclei, blue), CC-3 (apoptosis, green), Beclin 1 (autophagy, white) and Ki67 (proliferation, pink). Scale bar = 100 μm. N = 3 athymic nude mice were used per CDX model tumor generation (two tumors/mouse) for *in vivo* gemcitabine sensitivity assessment; n = 1 athymic mouse was used per cell line for TE tissue generation.

Gemcitabine sensitivity of AsPc-1, PANC-1 and Capan-1 xenografts was quantified by tumor volume measurements during the course of treatment *in vivo* that mirrored clinical dosing schedules. A positive treatment response was observed for the PANC-1 xenograft cohort, where decreased tumor size was seen over the therapy course. Limited response was seen in the AsPC-1 xenograft cohort with a slight tumor volume decrease in the treated vs. control group. No treatment response was seen in the Capan-1 xenograft cohort and overall tumor volume was highly variable (Fig 3A–3C). A possible mechanism for this response may be explained by gemcitabine uptake differences potentiated by differences in human equilibrative nucleoside transporter 1 (hENT1) receptor expression levels, where hENT1 is required for gemcitabine uptake. IF quantification of hENT1 expression in the established PDAC cell lines demonstrated high to low expression levels (PANC-1>AsPC-1>Capan-1) indicative of selective therapeutic uptake in the gemcitabine responsive cell line (S1 Fig).

Gemcitabine treatment response in explant culture from the same three xenografted cell lines was assessed following 48 hours of treatment. Tumor explants were administered a single gemcitabine dose to determine the utility of the tumor explant assay for prediction of therapeutic efficacy vs. matched tumors grown *in vivo*, where explant culture could provide rapid prediction capabilities for personalized medicine within a week of tumor biopsy or resection. Gemcitabine responses were similar in explant culture to the *in vivo* model systems with minimal response in the AsPC-1 tumors, notable response in the PANC-1 tumors and variable response in the Capan-1 tumors as measured by decrease in proliferation (Ki67) and increase in apoptosis (CC-3, Fig 3D–3F). Representative examples of tissues staining with the antibody viability panel (Ki67, CC-3 and Beclin 1) qualitatively demonstrated similar gemcitabine response profiles to those determined through quantitative *in vivo* tumor volume measurements or explant image analysis. This further supports the gemcitabine sensitivity levels of each CDX model (Fig 3G). Vehicle treated samples were included in the analysis, and displayed a minimal response to the gemcitabine treatment, as expected.

**Fig 4:**
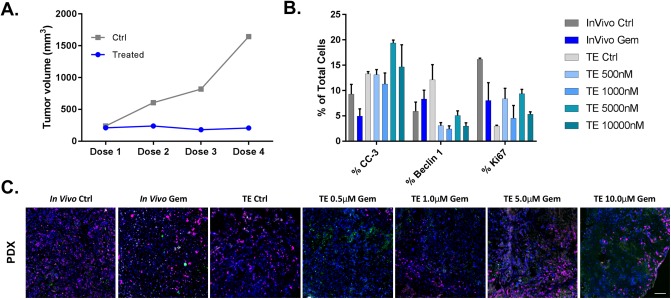
Gemcitabine (Gem) treatment equivalence in a sensitive PDX xenograft model *in vivo* vs. *ex vivo*. Mice bearing xenografts established from a gemcitabine sensitive patient tissue were treated with 100 mg/kg gemcitabine IP on days 0, 3, 6 and 9 to model clinical dose and administration schedule. **A**. Tumor volumes were quantified before and during treatment for both vehicle (Ctrl) and Gem treated PDX models. *Ex vivo* explants (TE) grown from untreated PDX tumors were treated with varied concentrations of gemcitabine (0.5–10 μM) for 48 hours. All resected tumor tissue was fresh frozen following the final dose of gemcitabine. **B**. Serially sectioned tissues were stained with the IF viability panel to assess biological response, where at least two 10 μm sections per condition were quantified for the percentage of positive cells. **C**. Representative IF viability panel images from untreated and gemcitabine treated tissues are shown with staining, false colored as DAPI (all nuclei, blue), CC-3 (apoptosis, green), Beclin 1 (autophagy, white) and Ki67 (proliferation, pink). Scale bar = 100 μm. N = 3 NSG mice were used for *in vivo* gemcitabine experiments; n = 1 NSG mouse was used for TE tumor generation.

To evaluate the utility of the explant model system for future clinical translation, studies were repeated on matched PDX models *in vivo* and as explant tumor culture. The patient tissue sample used for this work was obtained from a PDAC patient that was responsive to the clinical gemcitabine therapy regimen. Excitingly, *in vivo* tumor volume measurements showed gemcitabine response of the PDX tumor as compared to control, untreated samples, supporting the selected murine gemcitabine treatment regimen as clinically relevant (Fig 4A). Similar to the gemcitabine sensitive PANC-1 tumor explants, the PDX tumor explants also showed decreased proliferation (Ki67) and increased apoptosis with increasing gemcitabine treatment concentrations (Figs 3E, 3G, 4B and 4C) further supporting the idea that explant culture can be used as a rapid assay to predict therapeutic efficacy and aid in personalized cancer therapy selection.

### Selection of optimal fluorophore for gemcitabine labeling

**Fig 5:**
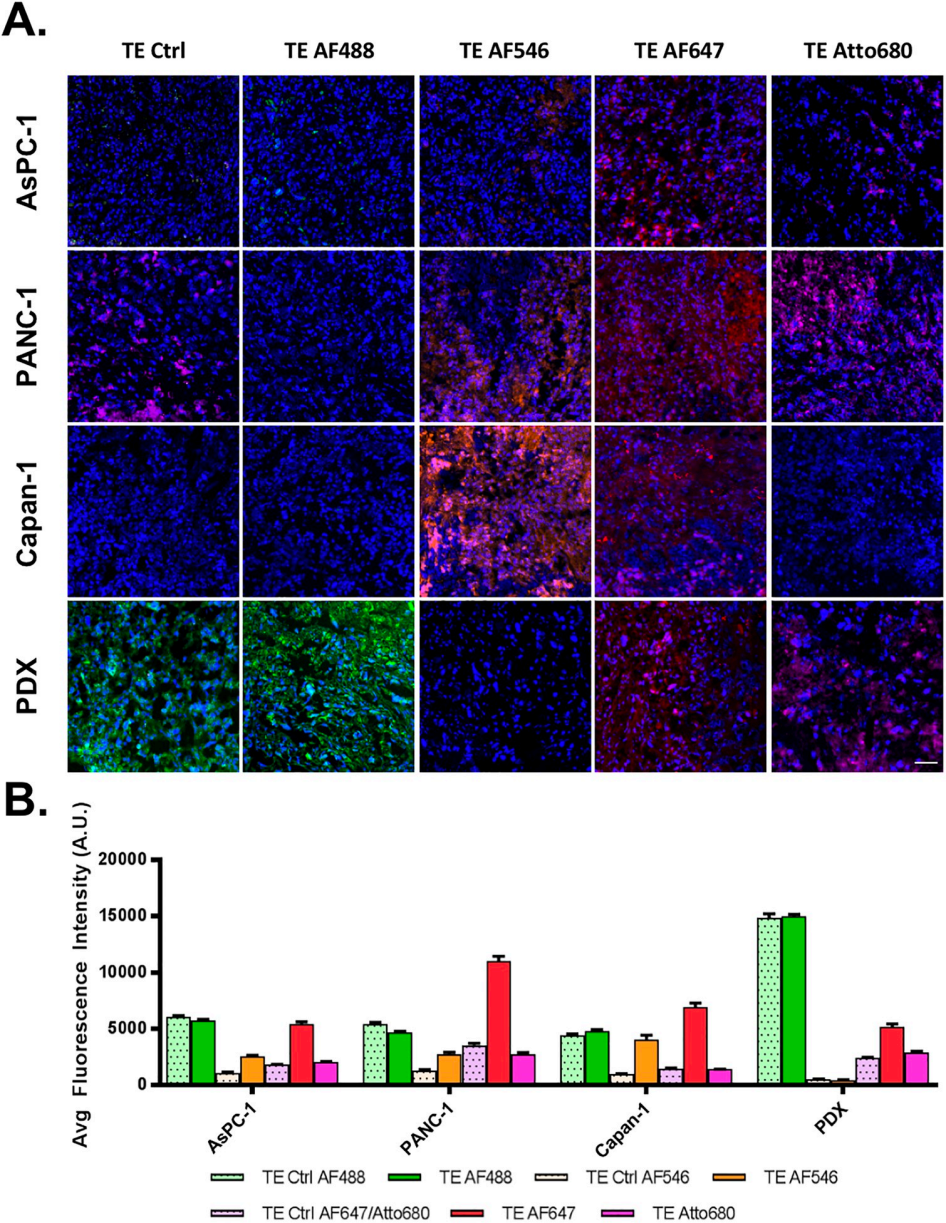
Small molecule fluorophore uptake and distribution in tumor explants (TE). Resected xenograft tissue grown as explants were incubated for 48-hour with 500 nM of nonreactive fluorophore representing different chemical scaffolds: AF488, AF546, AF647 and Atto680. **A**. Representative images from fresh frozen samples, shown normalized between mouse models within each respective fluorophore channel, demonstrated relatively homogenous distribution of fluorescence signal throughout the explant (TE). Control, unstained tissue was imaged in each channel to quantify autofluorescence (Ctrl). Scale bar = 50 μm. **B**. A minimum of ten 10 μm sections per fluorophore were quantified for average fluorescence intensity for each respective channel to evaluate non-specific fluorophore uptake in explant culture, where non-specific fluorophore uptake was the lowest for Atto680. N = 3 athymic nude mice were used per CDX model (two tumors/mouse) for TE tissue generation. Likewise, n = 1 NSG mouse was used to provide sufficient TE tumor material for all PDX fluorophore uptake experiments.

In addition to selection of personalized medicine, tumor explant culture also provides the opportunity to evaluate drug distribution using fluorescently labeled therapeutics in clinically relevant samples to aid in understanding of response and resistance mechanisms to therapy. Studies were conducted to select the optimal fluorophore for fluorescently labeled drug studies, where selection criteria were based on limiting non-specific uptake and biodistribution, resulting in minimal fluorescence signal of the parent fluorophore incubated explant tissues. Four commonly-used, commercially available fluorophores were selected for evaluation based on fluorophore family, overall net charge and color including AF488 (xanthene fluorophore family, net negative charge), AF546 (xanthene fluorophore family, net negative charge), AF647 (cyanine fluorophore family, net negative charge) and Atto680 (oxazine fluorophore family, net neutral charge). Tumor explant tissues that were incubated with 500 nM of each fluorophore for 48 hours displayed markedly different uptake profiles, confirming our hypothesis that fluorophore choice is critical for successful fluorescent labeling of drugs. Notably, all fluorophores showed roughly equivalent distribution throughout the tumor explant (Fig 5A), with no noticeable increased accumulation in necrotic regions (S2 Fig). As expected, high autofluorescence was observed in control tissues imaged using equivalent parameters to AF488 treated tissues, where incubation with 500 nM AF488 barely increased overall signal. Interestingly, PDX samples had intrinsically higher autofluorescence in this channel compared to CDX samples, further diminishing any utility of fluorescent labeling at these wavelengths (Fig 5B). Extensive fluorophore accumulation was observed for both AF546 and AF647, both negatively charged fluorophore scaffolds, resulting in substantially increased fluorescence in fluorophore treated explants compared to control autofluorescence. Conversely, the zwitterionic, cell membrane permeable oxazine fluorophore, Atto680, showed minimal uptake in explant culture even after a 48-hour incubation with 500 nM fluorophore. Thus, Atto680 was selected as the optimal compound for gemcitabine labeling since any accumulated fluorescence signal could be attributed to gemcitabine accumulation rather than non-specific background signal, as would be the case for either AF546 and AF647 (Fig 5).

### Tumor explant vs. *in vivo* fluorophore uptake

Gemcitabine was fluorescently labeled with Atto680 at the 4-amino group to minimize changes to therapeutic efficacy, yielding the fluorescently labeled version termed Gem-Atto (Fig 1). *In vitro* competitive binding studies comparing the parent drug with labeled gemcitabine showed a decreasing trend in fluorescence intensity with increased unlabeled gemcitabine concentration co-incubation in the gemcitabine sensitive PANC-1 cell lines, although variability was high (S3 Fig). No consistent trend in fluorescence was seen in the AsPC-1 and Capan-1 cell line competitive binding studies, which could be attributed to the lower hENT1 expression levels in these cell lines as compared to PANC-1 (S1 and S3 Figs). Overall these results support our hypothesis that fluorophore labeling at the 4-amino group of gemcitabine maintains the ability of the drug to incorporate into the DNA at a similar rate to the unlabeled, parent drug, preserving the drug’s therapeutic efficacy even in its fluorescently labeled state.

**Fig 6:**
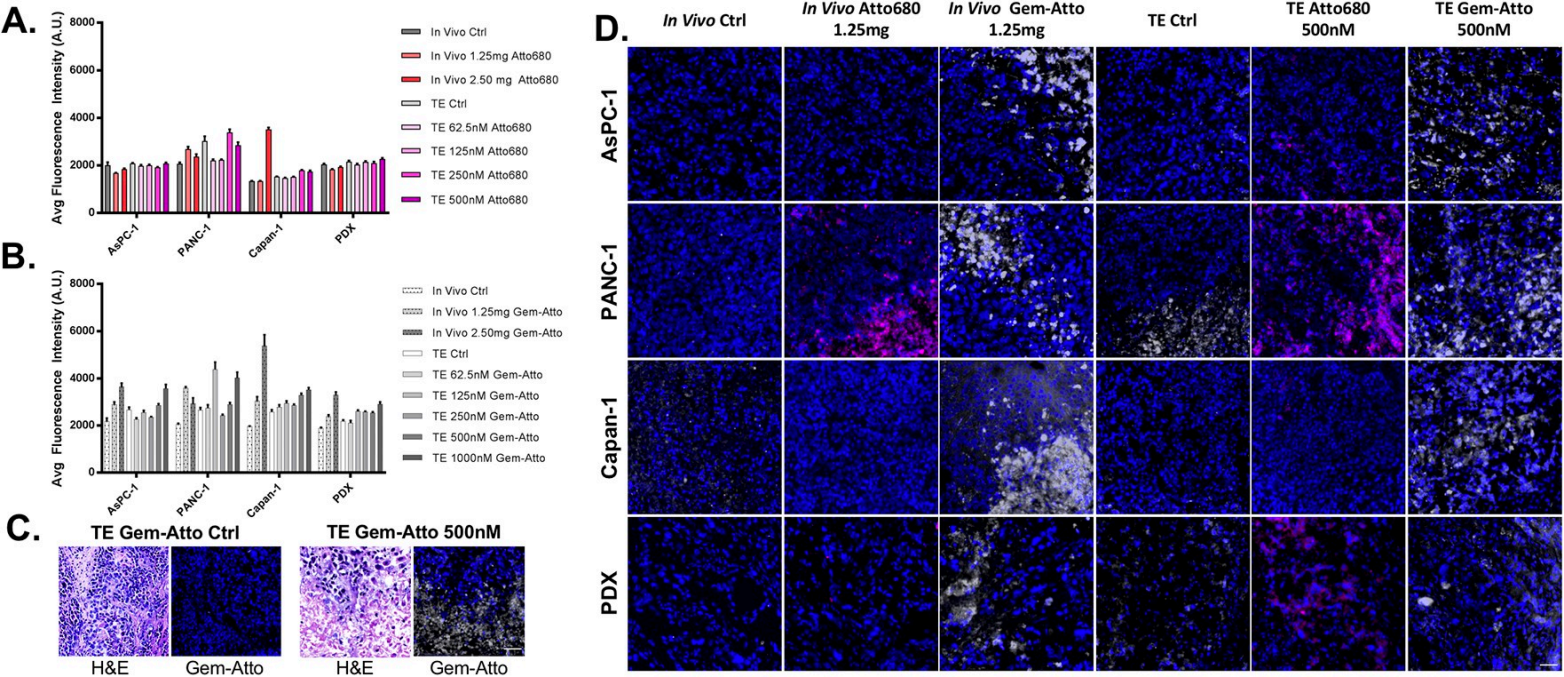
Gemcitabine-Atto680 (Gem-Atto) and parent fluorophore (Atto680) administration. **A**. Tumor explant (TE) tissues were subjected to increasing concentrations of Atto680 (62.5-500nM) in optimized media for 48 hours, while mice were administered Atto680 (1.25–2.50mg) for 4 hours prior to tissue collection. Average tissue fluorescence was quantified and compared across model systems. **B**. Tumor explants and *in vivo* tissues were also subjected to various concentrations of the drug-fluorophore construct, Gem-Atto. **C**. Representative Capan-1 H&E and corresponding Gem-Atto fluorescence images for both control and treated tumor explant samples, illustrated localized uptake in necrotic regions. **D**. Representative tissue ROIs showed distinct fluorescence intensity and spatial distributions in the explants versus *in vivo* samples for the parent fluorophore (Atto680, pink) and Gem-Atto (gray). Scale bar = 50 μm. N = 6 athymic nude mice were used per CDX (two tumors/mouse) for *in vivo* Atto680 and Gem-Atto assessment, while n = 5 athymic mice were used per cell line for TE tissue generation. Likewise, N = 6 NSG PDX mice were used for *in vivo* Atto680 and Gem-Atto experiments, while n = 1 NSG mouse was used for PDX TE tissue generation.

Tissues were subjected to varying titrations of Atto680 *in vivo* and *ex vivo* (62.5-500nM *ex vivo*, 1.25mg-2.50mg *in vivo*) followed by fluorescence intensity quantification, facilitating quantification of Gem-Atto uptake and distribution in matched tumor explant and *in vivo* tissues. Atto680 concentrations were chosen based on clinically relevant therapeutic concentrations, which were kept consistent throughout all remaining *in vivo* studies regardless of the administered reagent. Notably, all tumor model systems displayed similar fluorescence behaviors *in vivo* and *ex vivo* regardless of concentration (Fig 6A), again showing minimal fluorescence uptake and distribution over existing tissue autofluorescence.

This culminating work sought to investigate the uptake and distribution differences in Gem-Atto following *in vivo* vs. *ex vivo* administration to demonstrate the potential utility of tumor explants as a platform for understanding response and resistance mechanisms for personalized therapy. Given the minimal Atto680 uptake *in vivo* and *ex vivo*, any observed fluorescence patterns following administration of our synthesized Gem-Atto could be attributed to the drug’s accumulation and binding pattern in tissue. Tumor explants were subjected to increasing concentrations of Gem-Atto (62.5-1000nM), where fluorescence intensity was quantified compared to control, untreated tumor explant tissues and the Atto680 only titration experiments. As expected, the Gem-Atto samples displayed higher fluorescence intensity than Atto680 in both *in vivo* and tumor explant samples in all model systems, confirming specific uptake of the fluorescently labeled drug molecule. Overall, fluorescence intensity increased with increasing concentrations of administered Gem-Atto (Fig 6A and 6B). Interestingly, much lower concentrations of incubated Gem-Atto were required in the tumor explant media than for systemic *in vivo* administration to match fluorescence intensities (nM *ex vivo* vs. mM *in vivo*), highlighting an additional advantage to the explant platform for cost-effective screening of an array of fluorescently labeled therapeutics compared to traditional *in vivo* assays. Of interest, drug distribution was not uniform, revealing distinct localization of the drug in certain regions of the tumor tissue, where regions of high Gem-Atto uptake were often correlated to necrotic tissue morphology (Fig 6C and 6D).

Tumor explants are increasingly accepted as a novel platform for rapid therapeutic assessment enabling personalized medicine. These preliminary studies provide greater confidence for utilizing explants for studying therapeutic accumulation and engagement using fluorescently labeled drug derivatives. The primary goal of this work was to provide a comprehensive analysis of small molecule localization within the tumor explant model systems in the context of a standard of care chemotherapy, culminating in a proof-of-concept investigation of fluorescently conjugated gemcitabine (Gem-Atto). The tumor explant tissue recapitulated the fluorescence retention observed in matched *in vivo* counterparts, suggesting that the tumor explant biology is representative of the small molecule tissue interaction *in vivo*. Together, these experiments illustrate the utility of tumor explants to serve as a platform for small molecule drug-tissue interaction studies. Further experiments seek to understand the biological reason for gemcitabine resistance in human patient tissue samples as it relates to spatial uptake and accumulation patterns of the drug visible using our novel fluorescently labeled conjugates. Our tumor explant culture studies are readily expandable to other therapeutics, combination drug therapies and cancer types, which will be investigated in future studies.

## Supporting information

S1 Fig hENT1 PDAC cell line characterization. Human pancreatic cancer cell lines: PANC-1, AsPC-1 and Capan-1 were stained using indirect immunofluorescence to quantify hENT1 expression. Fluorescence microscopy was completed and cellular fluorescence was quantified on an individual cell basis permitting plotting of the mean and SEM per cell line.
(PDF)

S2 Fig Evaluation of tumor vs. necrotic fluorophore uptake in tumor explant (TE) tissues. Fluorescence intensity quantification per tissue was assessed based on uptake in viable tumor vs. necrotic tissue regions, as determined by H&E staining. No statistically significant difference in fluorophore uptake was detected between the viable tumor and necrotic tissues in any of the model systems.
(PDF)

S3 Fig Gem-Atto competitive binding studies. Human pancreatic cancer cell lines: PANC-1, AsPC-1 and Capan-1 were co-incubated with parent gemcitabine (0, 2.5, 25, 250, 2500, 25000 nM) and fluorescently labeled Gem-Atto (500 nM) for 24 hours to ensure cellular incorporation. Single-cell Gem-Atto fluorescence intensities were imaged via fluorescence microscopy and quantified.
(PDF)
