# Supplementary material for: Quantification of fluorophore distribution and therapeutic response in matched in vivo and ex vivo pancreatic cancer model systems
Source: PLoS One. 2020 Feb 25;15(2):e0229407. doi: 10.1371/journal.pone.0229407 (PMC7041865; doi:10.1371/journal.pone.0229407)
Supplement: S1 Fig — Human pancreatic cancer cell lines: PANC-1, AsPC-1 and Capan-1 were stained using indirect immunofluorescence to quantify hENT1 expression. Fluorescence microscopy was completed and cellular fluorescence was quantified on an individual cell basis permitting plotting of the mean and SEM per cell line. (PDF) [file pone.0229407.s001.pdf]

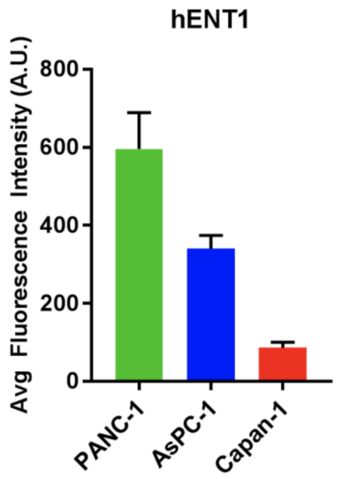

S1 Fig. hENT1 PDAC cell line characterization. Human pancreatic cancer cell lines: PANC-1, AsPC-1 and Capan-1 were stained using indirect immunofluorescence to quantify hENT1 expression. Fluorescence microscopy was completed and cellular fluorescence was quantified on individual cells permitting plotting of the mean and SEM per cell line.
