# Supplementary material for: Quantification of fluorophore distribution and therapeutic response in matched in vivo and ex vivo pancreatic cancer model systems
Source: PLoS One. 2020 Feb 25;15(2):e0229407. doi: 10.1371/journal.pone.0229407 (PMC7041865; doi:10.1371/journal.pone.0229407)
Supplement: S2 Fig — Fluorescence intensity quantification per tissue was assessed based on uptake in viable tumor vs. necrotic tissue regions, as determined by H&E staining. No statistically significant difference in fluorophore uptake was detected between the viable tumor and necrotic tissues in any of the model systems. (PDF) [file pone.0229407.s002.pdf]

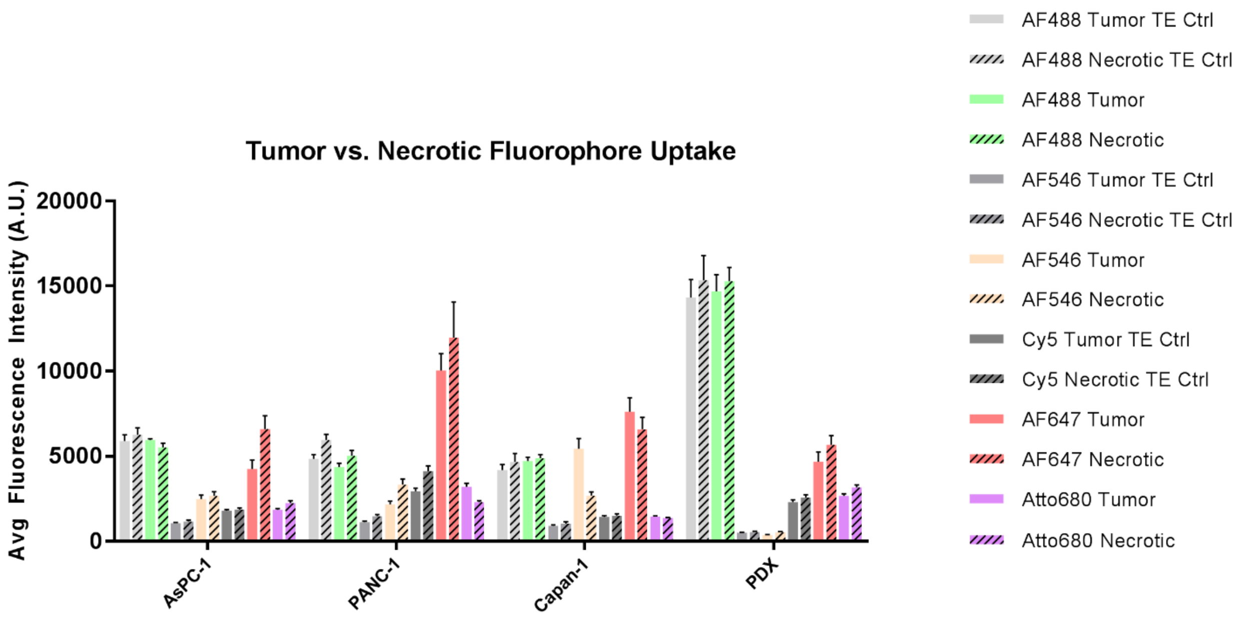

S2 Fig. Evaluation of tumor vs. necrotic fluorophore uptake in tumor explant (TE) tissues. Fluorescence intensity quantification per tissue was assessed based on uptake in tumor vs. necrotic tissue regions, as determined by H&E staining. No statistically significant difference in fluorophore uptake was detected between the normal tumor and necrotic tissues in any of the model systems.
