# Supplementary material for: Quantification of fluorophore distribution and therapeutic response in matched in vivo and ex vivo pancreatic cancer model systems
Source: PLoS One. 2020 Feb 25;15(2):e0229407. doi: 10.1371/journal.pone.0229407 (PMC7041865; doi:10.1371/journal.pone.0229407)
Supplement: S3 Fig — Human pancreatic cancer cell lines: PANC-1, AsPC-1 and Capan-1 were co-incubated with parent gemcitabine (0, 2.5, 25, 250, 2500, 25000 nM) and fluorescently labeled Gem-Atto (500 nM) for 24 hours to ensure cellular incorporation. Single-cell Gem-Atto fluorescence intensities were imaged via fluorescence microscopy and quantified. (PDF) [file pone.0229407.s003.pdf]

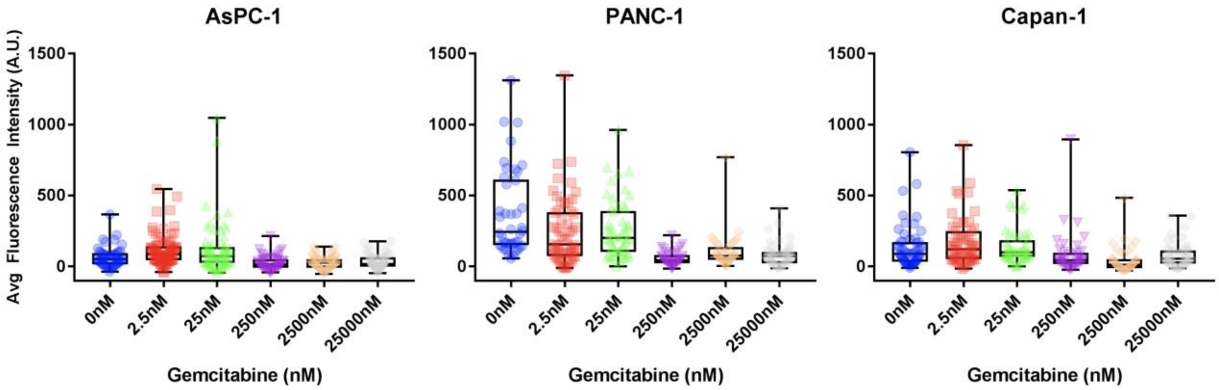

S3 Fig. Gem-Atto competitive binding studies. Human pancreatic cancer cell lines: PANC-1, AsPC-1 and Capan-1 were co-incubated with parent gemcitabine (0, 2.5, 25, 250, 2500, 25000nM) and fluorescently labeled Gem-Atto (500nM) for 24 hours to ensure cellular incorporation. Single-cell Gem-Atto fluorescence intensities were imaged via fluorescence microscopy and quantified.
